# Acquired *ROS1* Intragenic Rearrangements as a Resistance Mechanism in *EGFR*-Mutant Non-Small Cell Lung Cancer: A Case Series

**DOI:** 10.3390/curroncol33060311

**Published:** 2026-05-27

**Authors:** Po-Tsen Liu, Yi-Lin Chen, Wan-Li Chen, Chung-Liang Ho, Chun-Hui Lee

**Affiliations:** 1 Division of Hematology, Department of Internal Medicine, National Cheng Kung University Hospital, College of Medicine, National Cheng Kung University, Tainan 704, Taiwan; n109462@mail.hosp.ncku.edu.tw; 2 Molecular Diagnosis Laboratory, Department of Pathology, National Cheng Kung University Hospital, Tainan 704, Taiwan; emerald@ncku.edu.tw (Y.-L.C.); lili0805lili@hotmail.com (W.-L.C.); clh9@mail.ncku.edu.tw (C.-L.H.); 3 Institute of Clinical Medicine, College of Medicine, National Cheng Kung University, Tainan 704, Taiwan; 4 Department of Oncology, National Cheng Kung University Hospital, College of Medicine, National Cheng Kung University, Tainan 704, Taiwan

**Keywords:** non-small cell lung cancer (NSCLC), *EGFR* mutation, *ROS1* rearrangement, tyrosine kinase inhibitor (TKI) resistance

## Abstract

Lung cancer is a leading cause of global cancer mortality, with *EGFR* mutations serving as a primary therapeutic target. Although *EGFR* tyrosine kinase inhibitors (TKIs) are initially effective, acquired resistance inevitably develops. While *ROS1* rearrangements are well-known baseline drivers, they are exceptionally rare as acquired resistance mechanisms. We utilized next-generation sequencing (NGS) to identify a rare ROS1 intragenic rearrangement (exons 35–37) in three never-smoking women with *EGFR*-mutant lung adenocarcinoma following progression on *EGFR* TKIs. Clinical courses were heterogeneous: one patient achieved a durable partial response using combined osimertinib and crizotinib. A second patient, intolerant to dual TKI therapy due to QTc prolongation and grade 3 edemas, achieved a sustained partial response with platinum-pemetrexed chemotherapy. The third patient exhibited polyclonal resistance, including *EGFR* C797S and *TP53* mutations, with fatal central nervous system progression. In this three-patient case series, *ROS1* exon 35–37 RNA-level intragenic rearrangements were repeatedly detected after *EGFR*-TKI progression, suggesting a rare transcript-level alteration within heterogeneous resistance evolution. However, its biological significance, driver versus passenger role, and therapeutic relevance remain uncertain. Combined *EGFR* and *ROS1* inhibition may be considered in selected cases, but further validation is required.

## Simple Summary

We describe three women with *EGFR*-mutant lung cancer in whom a rare *ROS1* exon 35–37 RNA-level intragenic rearrangement was detected after progression on *EGFR*-targeted therapy. Our series demonstrates that combining two different targeted therapies to block both mutations can lead to long-term survival in one case, but another case may experience significant side effects. When combined therapy is not tolerated, traditional chemotherapy remains a necessary and effective alternative. This study highlights the potential value of advanced molecular testing after treatment failure and underscores the need for individualized treatment strategies and further functional validation when this specific *ROS1* rearrangement is detected.

## 1. Introduction

Lung cancer remains the leading cause of cancer-related mortality worldwide and ranks first in both incidence and mortality in Taiwan. Non-small cell lung cancer (NSCLC) accounts for approximately 85% of cases, with epidermal growth factor receptor (*EGFR*) mutations representing the most prevalent actionable driver, occurring in up to 40% of East Asian populations, particularly among women and never-smokers [1,2]. The most common alterations include exon 19 deletions and the exon 21 L858R point mutation [2].

*ROS1* rearrangements, first reported in 2007, occur in fewer than 2% of NSCLC cases globally and approximately 3% in East Asian populations [3,4]. Traditionally, *EGFR* mutations and *ROS1* rearrangements have been considered mutually exclusive oncogenic drivers. However, with the widespread adoption of next-generation sequencing (NGS), rare cases harboring concurrent alterations have been increasingly recognized, with reported prevalence ranging from 0.7% to 3.2% [5,6]. Most reported cases describe coexisting *EGFR* and *ROS1* alterations at baseline, whereas acquired *ROS1* rearrangements following *EGFR*-targeted therapy remain exceedingly uncommon.

We present three never-smoking women with *EGFR*-mutant lung adenocarcinoma who developed acquired *ROS1* intragenic rearrangements involving exons 35–37 detected by NGS (detailed methods are provided in Appendix A) after *EGFR* tyrosine kinase inhibitor (TKI) exposure, demonstrating heterogeneous clinical courses and therapeutic responses.

## 2. Case Presentation

### 2.1. Case 1: Durable Disease Control After Dual Targeted Therapy (Summarized in Figure 1)

A 50-year-old never-smoking female with lung adenocarcinoma harboring an EGFR exon 21 p.L858R mutation presented with stage IIIB disease (cT4N2M0, American Joint Committee on Cancer, 8th edition) with mediastinal lymph node involvement deemed surgically unresectable. Standard concurrent chemoradiotherapy was recommended at the outside hospital; however, the patient declined this treatment plan, and first-line afatinib (30 mg daily) was initiated after shared decision-making. After transferring care to our institution, chest computed tomography (CT) at seven months of afatinib therapy demonstrated a 2.8 cm left upper lobe (LUL) mass with progressive mediastinal lymphadenopathy. Transbronchial needle aspiration revealed no malignancy, and afatinib was continued. At 13 months, CT showed significant progression with LUL tumor enlargement, pleural seeding, massive left pleural effusion, and extensive lymphadenopathy involving mediastinal, paraoesophageal, bilateral supraclavicular, para-aortic, aortocaval, and retrocaval regions. CT-guided biopsy of the LUL mass confirmed adenocarcinoma without histological transformation. Comprehensive NGS revealed the persistence of the *EGFR* L858R mutation, along with a *ROS1* exon 35–37 RNA-level intragenic rearrangement transcript (Supplementary File S1). Significantly, no concurrent T790M mutation or *MET* amplification was detected. Following 14 months of afatinib, combination therapy with osimertinib (80 mg daily) and crizotinib (250 mg twice daily) was initiated. The patient tolerated treatment well, experiencing only grade 1 transaminitis. CT at three months demonstrated marked tumor response with significant reduction in primary tumor size, near-complete resolution of pleural effusion and nodules, and substantial nodal regression. To date, the patient continues dual therapy with a sustained clinical and radiographic response exceeding 32 months from the initiation of the initial afatinib treatment and 18 months from the initiation of combination therapy.

**Figure 1:**
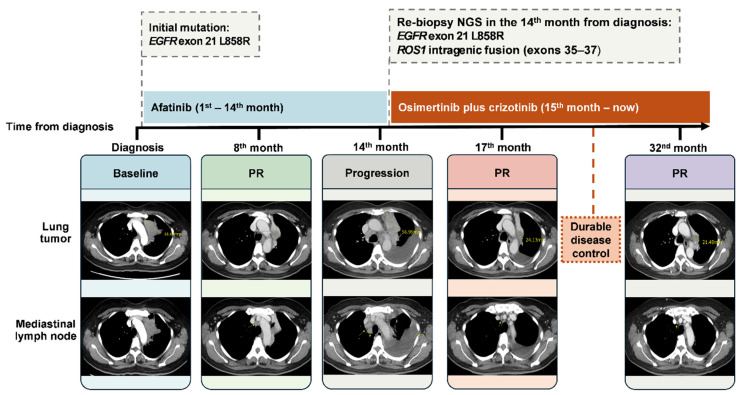
Timeline of Case 1 with durable response to dual *EGFR*-*ROS1* inhibition in *EGFR*-mutant NSCLC with acquired *ROS1* rearrangement. Serial chest CT images show changes in the target lung lesion and mediastinal lymph nodes. The lung tumor measured 62 mm at diagnosis, decreased to 29 mm at month 8, increased to 57 mm at progression at month 14, and decreased to 24 mm and 21 mm after osimertinib plus crizotinib at months 17 and 32, respectively. Target lesion measurements and response assessments are shown according to RECIST v1.1 where evaluable. PR: partial response; NGS: next-generation sequencing.

### 2.2. Case 2: Dual-TKI Intolerance Followed by Chemotherapy (Summarized in Figure 2)

A 55-year-old never-smoking woman with hypertension presented with progressive dyspnea, malaise, and reduced appetite. Chest CT revealed a 3.1 cm spiculated LUL mass with extensive bilateral hilar, mediastinal, and supraclavicular lymphadenopathy, bilateral pleural effusions, and pericardial effusion. CT-guided lung biopsy confirmed moderately differentiated adenocarcinoma. Cytology demonstrated malignant cells in pericardial and pleural effusions. Bone scintigraphy revealed sternal metastasis; brain magnetic resonance imaging (MRI) was negative (stage cT2aN3M1b, IVA, American Joint Committee on Cancer, 8th edition). Polymerase chain reaction identified *EGFR* exon 21 p.L858R and *PIK3CA* p.E545K mutations. The patient initiated gefitinib (250 mg daily) with concurrent palliative radiotherapy (30 Gy in 10 fractions) to bulky mediastinal nodes pending government National Health Insurance (NHI) approval for afatinib reimbursement. Three weeks later, treatment transitioned to afatinib (30 mg daily) with bevacizumab (15 mg/kg every 3 weeks), completing seven cycles over five months. CT at three months showed partial response with reduced tumor size and pleural effusion. However, two months later, pleural effusion worsened with tumor enlargement on radiography. Pleuroscopic biopsy confirmed metastatic adenocarcinoma. CT at six months demonstrated progressive disease per RECIST v1.1: LUL tumor enlargement to 3.4 cm, new pleural seeding, progressive lymphadenopathy, and contralateral pulmonary nodules. Repeat NGS revealed persistent *EGFR* L858R, persistent *PIK3CA* E545K, and newly detected *ROS1* RNA-level intragenic rearrangement transcript of exons 35–37 (Supplementary File S1). *EGFR* T790M and *MET* amplification were absent. A combination of osimertinib (80 mg daily) and crizotinib (250 mg twice daily) was initiated five months after afatinib. However, QTc prolongation to 548 ms (baseline: 412 ms) after 6 days of dual therapy prompted discontinuation of osimertinib. Crizotinib was subsequently discontinued due to grade 3 lower-extremity edema refractory to diuretics. Given dual TKI intolerance, the patient transitioned to pemetrexed (500 mg/m^2^) plus carboplatin (AUC 5), completing six cycles over 18 weeks with manageable grade 1–2 toxicities. CT after four cycles demonstrated partial response with regression of LUL mass, bilateral pulmonary metastases, pleural and pericardial disease, and nodal burden per RECIST v1.1. Maintenance pemetrexed 500 mg/m^2^ was initiated; CT after three cycles confirmed a sustained partial response. The patient has received five maintenance cycles, surviving 13 months since diagnosis with a sustained response for seven months following chemotherapy initiation.

**Figure 2:**
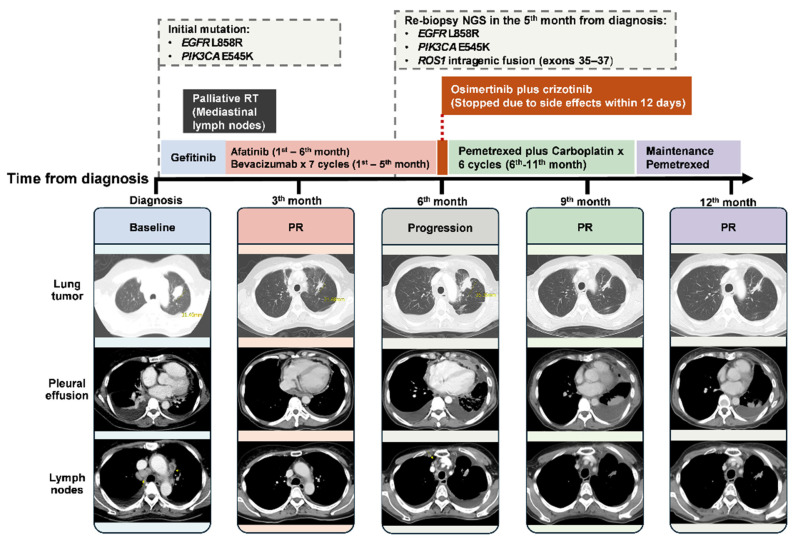
Timeline of Case 2 with successful platinum-based chemotherapy salvage following dual TKI intolerance in *EGFR*-mutant NSCLC with acquired *ROS1* rearrangement. Serial chest CT images show changes in the target lung lesion, pleural effusion, and lymph nodes. The lung tumor measured 31 mm at diagnosis, decreased to 22 mm at month 3, increased to >35 mm at progression at month 6, and decreased to 17 mm and 16 mm after platinum-pemetrexed chemotherapy at months 9 and 12, respectively. Target lesion measurements and response assessments are shown according to RECIST v1.1 where evaluable. PR: partial response; NGS: next-generation sequencing.

### 2.3. Case 3: Polyclonal Resistance with CNS Progression (Summarized in Figure 3)

A 68-year-old never-smoking woman with hypertension and dyslipidemia presented with progressive left arm weakness. Brain MRI revealed multiple cerebral and cerebellar metastases. Chest CT demonstrated a 4.6 cm lower lobe mass with contralateral mediastinal lymphadenopathy, intrathoracic metastases, and extensive osseous involvement. Lung biopsy confirmed adenocarcinoma harboring an *EGFR* exon 19 deletion (stage cT4N3M1c, IVB, American Joint Committee on Cancer, 8th edition). Osimertinib (80 mg daily) was initiated with concurrent palliative radiotherapy to symptomatic bone metastases (30 Gy in 10 fractions). Serial imaging over 17 months demonstrated durable systemic and intracranial disease control. At 19 months, the patient developed progressive right-sided weakness. Brain MRI showed progression of multiple brain metastases. Left frontoparietal craniotomy with gross total resection was performed. NGS of the resected brain lesion revealed a persistent *EGFR* exon 19 deletion, an acquired *EGFR* C797S mutation, a *TP53* p.R248W mutation, and *a ROS1* exon 35–37 RNA-level intragenic transcript event. (Supplementary File S1). On postoperative day 2, the patient developed ischemic stroke with hemorrhagic transformation, requiring emergent decompressive craniectomy and hematoma evacuation. She remained obtunded, with an ECOG performance status of 4. Given the poor prognosis, the patient and family declined further systemic therapy. She was transitioned to palliative care and died approximately 20 months from diagnosis.

**Figure 3:**
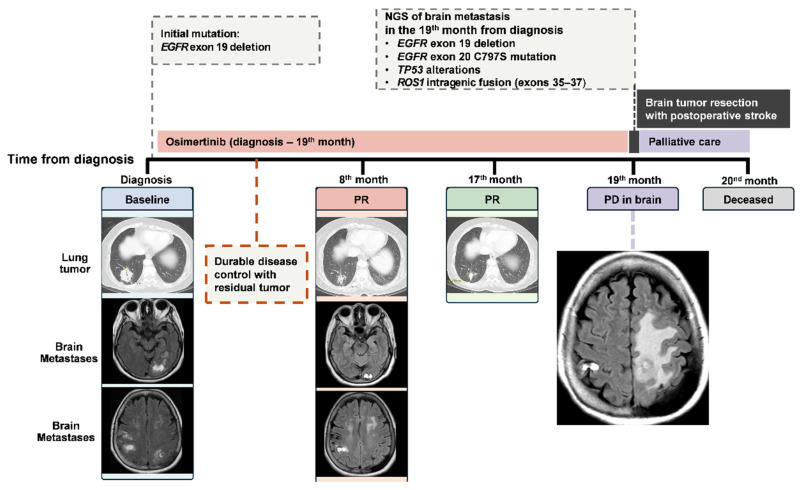
Timeline of case 3 with polyclonal resistance and CNS progression in *EGFR*-mutant NSCLC with acquired *EGFR* C797S and *ROS1* rearrangement. Serial chest CT and brain MRI images show systemic response followed by CNS progression. The lung tumor measured 46 mm at diagnosis, decreased to 33 mm at month 8, and further decreased to 26 mm at month 17 during osimertinib therapy. Brain progression occurred at month 19. Target lung lesion measurements and response assessments are shown according to RECIST v1.1 where evaluable. PR: partial response; PD: progressive disease; NGS: next-generation sequencing.

## 3. Discussion

This case series describes the repeated detection of *ROS1* exon 35–37 RNA-level intragenic transcript events after *EGFR*-TKI progression in *EGFR*-mutant NSCLC (Table 1). In *EGFR*-mutant NSCLC, acquired resistance most commonly arises from on-target *EGFR* alterations, particularly the C797S mutation, which occurs in approximately 15% of patients following osimertinib [7]. *EGFR*-independent resistance mechanisms include bypass pathway activation through *MET* amplification, *HER2*, *BRAF*, or *KRAS* alterations [8,9]. The *ROS1* exon 35–37 RNA-level transcript events observed in this series may represent rare resistance-associated alterations within this broader landscape; however, their biological significance and driver versus passenger role remain uncertain, and further orthogonal and functional validation is required.

Recent NGS-based analyses have shown that acquired resistance after *EGFR*-TKI therapy is molecularly heterogeneous, involving both on-target *EGFR* alterations and off-target bypass mechanisms. In the AURA3 resistance analysis, Chmielecki et al. reported multiple acquired genomic alterations after second-line osimertinib, including *MET* amplification and *EGFR* C797X, with some patients harboring more than one resistance-related alteration [10]. In addition, Xia et al. showed that acquired receptor tyrosine kinase fusions may represent rare bypass resistance events after *EGFR*-TKI exposure, supporting the broader concept of fusion-driven resistance biology [11]. Acquired *ROS1* alterations remain exceptionally rare. In a multicenter retrospective analysis of 27 patients with *EGFR*-mutant NSCLC who developed acquired gene fusions following *EGFR*-TKI progression, *ROS1* rearrangements were observed in only two patients (7.4%), and acquired *ROS1* fusions accounted for only 1.3% of all reported fusion events [12]. The *ROS1* proto-oncogene, located on chromosome 6q22.1, consists of 43 exons, with the tyrosine kinase domain encoded by exons 36–41. Most recurrent *ROS1* fusions result from interchromosomal translocations demonstrating ligand-independent catalytic kinase activity, with *CD74*–*ROS1* representing the most frequent fusion partner [13]. Notably, all three cases in our series shared a rare *ROS1* intragenic rearrangement transcript involving exons 35–37, identified by an RNA-based fusion panel. Unlike canonical *ROS1* fusions involving well-characterized partners such as *CD74* or *SLC34A2*, intragenic rearrangements are poorly understood and lack functional validation in most reports. While definitive conclusions remain premature, the temporal co-occurrence of these alterations with *EGFR*-TKI resistance—alongside the clinical response observed following *ROS1*-directed therapy in one patient—warrants further investigation into their potential biological and therapeutic implications.

Currently, no established standard treatment recommendations exist for patients harboring concurrent *EGFR* mutations and acquired *ROS1* rearrangements. Drawing on experience from strategies targeting acquired *MET* amplification following osimertinib resistance—notably in the TATTON, SAVANNAH, and INSIGHT-2 trials—combination therapies directed against both *EGFR* and the acquired receptor tyrosine kinase alteration have demonstrated meaningful response durations with acceptable toxicity profiles [14,15,16].

In our first case, dual therapy with osimertinib and crizotinib led to marked tumor regression and durable disease control for nearly three years (Figure 1). Following progression on afatinib without a detectable *EGFR* T790M mutation, osimertinib was initiated based on clinical evidence suggesting potential efficacy in T790M-negative populations. Data from the TREM study reported an overall response rate of 28% and a median progression-free survival (PFS) of 5.1 months in T790M-negative patients [17]. Similarly, the WJOG12819L study demonstrated a median PFS of 4.07 months [18], while retrospective analysis by Cheng et al. indicated a median PFS of 10.2 months with third-generation TKIs in this subgroup [19]. Notably, our patient’s sustained response exceeding 18 months on dual therapy significantly surpasses these historical medians. Nevertheless, evidence supporting dual TKI therapy for acquired *ROS1* rearrangements remains limited to case reports. One report described a 48-year-old female with *EGFR* exon 19 deletion and an acquired *SLC32A2*–*ROS1* fusion who achieved a favorable response with combined furmonertinib and crizotinib [20]. Another case involved a 60-year-old female with *EGFR* exon 19 deletion who developed a *GOPC*–*ROS1* (G8:R35) rearrangement at second progression. Treatment with osimertinib and crizotinib resulted in a partial response with manageable adverse events limited to grade 2 rash and diarrhea. Previously reported adverse events associated with this combination include serum creatinine elevation, pneumonitis, diarrhea, and fatigue [21]. Notably, based on our Case 2, QTc prolongation and potential cardiac arrhythmia risk should be carefully monitored in clinical practice.

In our second case, the patient achieved sustained partial response and durable disease stabilization with platinum–pemetrexed chemotherapy followed by pemetrexed maintenance (Figure 2). Given documented objective response rates (ORR) of approximately 80% with crizotinib in *ROS1* fusion-positive NSCLC compared to <50% with cytotoxic regimens, dual TKI therapy was initially attempted [22,23]. However, following the development of treatment-limiting toxicities, therapy was transitioned to a platinum-based backbone. The efficacy of chemotherapy in this context is underscored by historical benchmarks from the IMPRESS [24] and KCSG-LU12-13 trials [25], in which platinum-pemetrexed combinations after TKI progression yielded median PFS rates of 4.2 to 5.6 months. Our patient’s response duration of 7 months exceeds the established survival. This clinical outcome suggests that cytotoxic chemotherapy remains a reasonable salvage option after EGFR-TKI progression and dual TKI intolerance.

The third case illustrates the complexity of resistance evolution, with concurrent *EGFR* C797S, *TP53* alterations, and *ROS1* rearrangement in the setting of CNS-dominant progression (Figure 3). The *EGFR* C797S mutation prevents covalent binding of osimertinib and represents a well-established on-target resistance mechanism [7]. The *TP53* p.R248W hotspot mutation is associated with genomic instability and shorter *EGFR* TKI progression-free survival [26]. The coexistence of acquired *EGFR* C797S and *TP53* R248W suggests heterogeneous or polyclonal resistance evolution, making it difficult to determine whether the *ROS1* alteration contributed to drug resistance or CNS progression. Although CNS-penetrant *ROS1* inhibitors such as entrectinib or later-generation *ALK*/*ROS1* inhibitors (e.g., lorlatinib or repotrectinib) may theoretically improve intracranial disease control, poor performance status and postoperative complications precluded further systemic therapy in this patient.

The repeated detection of a *ROS1* exon 35–37 RNA-level transcript event across three cases is noteworthy. However, the mechanism underlying this transcript event remains unclear and may reflect heterogeneous resistance evolution under *EGFR*-TKI selective pressure, clonal selection of pre-existing minor populations, aberrant splicing, or a non-driving passenger event [27,28]. We propose an exploratory model in which *EGFR*-TKI exposure may be associated with diverse resistance patterns, including canonical resistance mechanisms and rare transcript-level alterations such as *ROS1* exon 35–37 rearrangement. A key limitation of this case series is that the temporal association between ROS1 exon 35–37 RNA-level transcript events and EGFR-TKI progression does not establish causality. Although these alterations may represent acquired resistance-associated events after EGFR-TKI exposure, alternative explanations, including passenger events or parallel evolutionary branches, cannot be excluded. Therefore, further functional studies are required to clarify their biological significance and potential therapeutic relevance.

This case series underscores several important clinical principles. First, tissue acquisition at progression remains critical when feasible, as molecular profiling can identify actionable resistance mechanisms and inform treatment decisions. Crucially, although the systematic integration of repeat NGS has yet to be established as the universal standard of care following *EGFR*-TKI failure, we strongly advocate for its routine clinical implementation. Conventional molecular assays may not fully capture rare or structurally complex resistance-associated alterations, particularly noncanonical RNA-level events such as the *ROS1* exon 35–37 transcript event observed in this series. Second, polyclonal resistance with multiple concurrent mechanisms is increasingly recognized with NGS-based profiling and requires a sophisticated combination of therapeutic approaches. Third, the recurrence of the same *ROS1* exon 35–37 rearrangement across three independent cases warrants further investigation. Finally, clinical circumstances, including performance status decline and treatment-related toxicities, may limit the ability to implement molecularly guided therapies, underscoring the importance of multidisciplinary evaluation and individualized treatment planning.

## 4. Conclusions

Albeit rare, *ROS1* exon 35–37 RNA-level intragenic transcript events were repeatedly detected after *EGFR*-TKI progression in three patients with clinically heterogeneous courses. The biological significance and driver versus passenger role of this alteration remain uncertain, highlighting the need for further orthogonal and functional validation. Combined *EGFR* and *ROS1* inhibition may be considered in selected cases, but its clinical utility requires further validation. Larger cohorts and functional studies are needed to clarify whether this rare transcript-level event contributes to *EGFR*-TKI.

## Figures and Tables

**Table 1:** Clinical characteristics and outcomes of three patients with *EGFR*-mutant NSCLC and acquired *ROS1* intragenic rearrangements.

Characteristic	Case 1	Case 2	Case 3
**Demographics**			
Age, years	50	55	68
Sex	Female	Female	Female
Smoking status	Never-smoker	Never-smoker	Never-smoker
**Disease Characteristics**			
Histology	Adenocarcinoma	Adenocarcinoma	Adenocarcinoma
Stage at diagnosis	IIIB (cT4N2M0)	IVA (cT2aN3M1b)	IVB (cT4N3M1c)
Baseline *EGFR* mutation	Exon 21 L858R	Exon 21 L858R	Exon 19 deletion
Co-mutations at baseline	None detected	*PIK3CA* E545K	Not reported
**Resistance Mechanisms**			
Time to progression on the first *EGFR* TKI, months	13	5	19
Acquired *ROS1* alteration (identified by the RNA Fusion XP Panel)	*ROS1* exon 35–37 intragenic rearrangement	*ROS1* exon 35–37 intragenic rearrangement	*ROS1* exon 35–37 intragenic rearrangement
Additional resistance mechanisms	None detected	None detected	*EGFR* C797S, *TP53* R248W
**Treatment Course**			
First-line *EGFR* TKI	Afatinib 30 mg daily	Gefitinib 250 mg daily → Afatinib 30 mg daily + Bevacizumab	Osimertinib 80 mg daily
Treatment at progression	Osimertinib 80 mg daily + Crizotinib 250 mg BID	Osimertinib 80 mg daily + Crizotinib 250 mg BID (discontinued due to toxicity)	None (declined)
Progression Pattern	systemic progression with pleural effusion and nodal progression	systemic progression with pleural/pericardial disease and pulmonary/nodal progression	CNS-dominant progression
Subsequent therapy	Ongoing dual TKI	Carboplatin-pemetrexed × 6 cycles → pemetrexed maintenance	Palliative care
**Clinical Outcomes**			
Best response to dual TKI	Marked tumor regression, with near-complete resolution of effusions	Not evaluable (discontinued day 12)	N/A
Adverse events	Grade 1 transaminitis	QTc prolongation (548 ms), grade 3 edema	N/A
Response to chemotherapy	N/A	Partial response, with sustained disease control	N/A

Abbreviations: BID, twice daily; CNS, central nervous system; *EGFR*, epidermal growth factor receptor; N/A, not applicable; NSCLC, non-small cell lung cancer; TKI, tyrosine kinase inhibitor.

## Data Availability

In accordance with ethical restrictions and patient privacy protocols, the data supporting this study are not publicly accessible. Inquiries regarding data access should be directed to the corresponding author.

## Supplementary Materials

The following supporting information can be downloaded at: https://www.mdpi.com/article/10.3390/curroncol33060311/s1, File S1: The sequencing of *ROS1* RNA-level intragenic rearrangement transcript of exon 35–37 in 3 cases.

## Abbreviations

The following abbreviations are used in this manuscript:

AJCC	American Joint Committee on Cancer
ALK	Anaplastic lymphoma kinase
AUC	Area under the curve
BID	Bis in die (Twice daily)
CNS	Central nervous system
CT	Computed tomography
ECOG	Eastern Cooperative Oncology Group
EGFR	Epidermal growth factor receptor
LUL	Left upper lobe
MET	Mesenchymal–epithelial transition
MRI	Magnetic resonance imaging
N/A	Not applicable
NGS	Next-generation sequencing
NHI	National Health Insurance
NSCLC	Non-small cell lung cancer
PD	Progressive disease
PR	Partial response
RECIST	Response Evaluation Criteria in Solid Tumors
TKI	Tyrosine kinase inhibitor

## Appendix A. The QIAseq Targeted DNA Pro Lung Cancer Research Panel and the QIAseq RNA Fusion XP Panel for Lung Cancer

We used the Lung Cancer Research Panel (cat. no. PHS-005Z; Qiagen GmbH) and the QIAseq RNA Fusion XP Panel to perform next-generation sequencing (NGS) on lung cancer cases. The DNA panel included the following lung cancer-related genes: ABL1, CTNNB1, MAP3K8, PTEN, BARD1, IRF1, PMS1, AKT1, DDR2, MET, RET, BRCA1, KIT, PMS2, ALK, EGFR, MYC, ROS1, BRCA2, MLH1, RAD51B, ATM, ERBB2, MYCN, SLC22A18, BRIP1, MLH3, RAD51C, BRAF, FGFR1, NRAS, STK11, CDK12, MSH2, RAD51D, CCND1, FGFR3, NTRK1, TP53, CHEK1, MSH3, RAD54L, CD274, HRAS, PIK3CA, TSC2, CHEK2, MSH6, RB1, CDK4, and KRAS. BAP1, FANCL, PALB2, CDKN2A, MAP2K1, PRKN, NF2, IDH1, IDH2, and PDGFRA. The RNA panel included the following driver genes: ALK, BRAF, RET, ROS1, RAF1, NTRK1, NTRK2, NTRK3, FGFR1, FGFR2, FGFR3, FGFR4, NRG1, EGFR, ERBB2, and MET. As well as diagnosis-related genes: AR, AXL, BAIAP2L1, BCOR, CCDC6, CCNB3, CD74, CIC, CLTC, CREB3L2, CRTC1, EML4, ERG, ETV1, ETV6, EWSR1, EZR, FLI1, FOXO1, FUS, GOPC, HIP1, KIF5B, KLC1, KMT2A (MLL), LMNA, LRIG3, MAML2, MLLT10, MYH9, NAB2, NACC2, NCOA4, NFATC2, NPM1, NUTM1, PDGFRA, PDGFRB, PIK3CA, PAX3, PRKAR1A, RANBP2, SDC4, SEPTIN14, SLC34A2, SLC45A3, SS18, SSX2, STAT6, STRN, TACC3, TFG, TMPRSS2, TP53, TPM3, TRIM33, and USP6. The QIAamp DNA FFPE Kit (Cat. No. 56404, Qiagen, Hilden, Germany) and the QIAGEN RNase FFPE Kit were used to purify FFPE DNA and RNA, respectively, for NGS. A total of 250 ng of FFPE DNA and 250 ng of FFPE RNA were used for library construction. The Lung Cancer Research Panel and the QIAseq RNA Fusion XP integrate unique molecular index (UMI) technology into a gene-specific primer-based target enrichment process. Construction of the DNA libraries followed the manufacturer’s instructions. Library quality control (QC) was performed using a QIAxcel DNA High Resolution Kit (cat. no. 929002; Qiagen GmbH) to verify the size distribution of the library. The final library’s loading concentration was 10 pM, as measured by a Qubit fluorometer. The sequencing run was performed using the MiSeq Reagent Kit v2 (cat. no. MS-102-2002). Paired-end libraries (2 × 150 bp) were sequenced using an Illumina MiSeq sequencer. Mean sequence depths of at least 500× were achieved for the tumor tissue. Data analysis was performed using CLC Genomics Workbench (version 24.0.1) and QIAGEN Clinical Insight (QCI) for variant interpretation. The data analysis focused on lung cancer-related genes using QIAGEN Clinical Insights Interpret software (version 9.4.2.20250513, QIAGEN GmbH) automatable interpretation workflows. The lung cancer research panel detection limit was a variant allele frequency (VAF) of at least 3%, and the lung RNA fusion XP panel detection limit was a fusion count of at least 5. The variants were classified as either pathogenic or likely pathogenic using QIAGEN Clinical Insight (QCI), which is a variant analysis, interpretation, and decision support tool for clinical laboratories that analyze NGS data.
