# Supplementary material for: Acquired ROS1 Intragenic Rearrangements as a Resistance Mechanism in EGFR-Mutant Non-Small Cell Lung Cancer: A Case Series
Source: Curr Oncol. 2026 May 27;33(6):311. doi: 10.3390/curroncol33060311 (PMC13298532; doi:10.3390/curroncol33060311)
Supplement: Supplementary file 1 [file curroncol-33-00311-s001.zip › curroncol-4286946-supplementary.pdf]

Fusion of *ROS1* Exon 35 and *ROS1* Exon 37

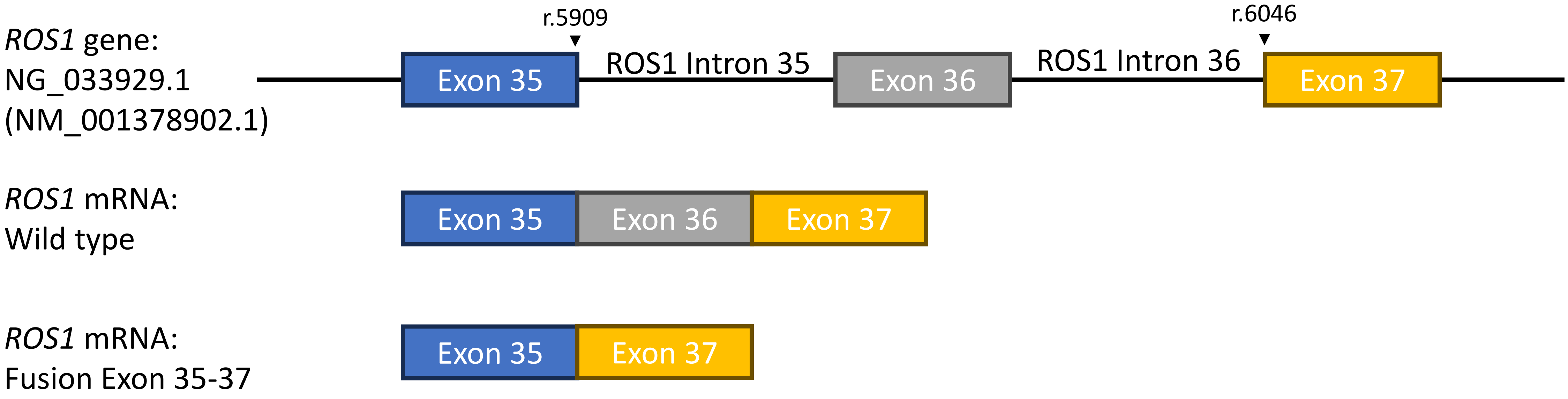

The molecular analysis reveals a non-canonical alternative splicing event within the *ROS1* gene, characterized by the skipping of Exon 36. BLAST sequence alignment against the reference transcript NM_001378902.1 identifies a direct fusion between the 3’ terminus of Exon 35 and the 5’ terminus of Exon 37. The breakpoint occurs at mRNA position r.5909, where the sequence immediately transitions to r.6046. This junction is consistently identified across multiple independent reads—Case 1, Case 2, and Case 3—validating the structural variant.

Case 1

TCTAGGGTTTGGTGAATATAGTGGAATCAGTGAGAATATTATATTAGTTGGAGATGATTTTTGGA
TACCAGAAACAAGTTTCATACTTACTATTATAGTTGGAATATTTCTGGTTGTTACAATCCCACTGAC
CTTTGTACTCTTCCAACCCAAGAGGAGATTGAAAATCTTCCTGCCTTCCCTCGGGAAAAACTGAC
TCTGCGTCTCTTGCTGG

Homo sapiens ROS proto-oncogene 1, receptor tyrosine kinase (ROS1), transcript variant 3, mRNA

Sequence ID: NM_001378902.1 Length: 8451 Number of Matches: 2

Range 1: 5773 to 5910 GenBank Graphics ▼ Next Match ▲ Previous Match

Score	Expect	Identities	Gaps	Strand
250 bits(276)	8e-70	138/138(100%)	0/138(0%)	Plus/Plus

```
Query  1     TCTAGGGTTTGGTGAATATAGTGGAATCAGTGAGAATATTATATTAGTTGGAGATGATTT  60
             ||||||||||||||||||||||||||||||||||||||||||||||||||||||||||||
Sbjct  5773  TCTAGGGTTTGGTGAATATAGTGGAATCAGTGAGAATATTATATTAGTTGGAGATGATTT  5832

Query  61    TTGGATACCAGAAACAAGTTTCATACTTACTATTATAGTTGGAATATTTCTGGTTGTTAC  120
             ||||||||||||||||||||||||||||||||||||||||||||||||||||||||||||
Sbjct  5833  TTGGATACCAGAAACAAGTTTCATACTTACTATTATAGTTGGAATATTTCTGGTTGTTAC  5892

Query  121   AATCCCACTGACCTTTG  138
             |||||||||||||||||
Sbjct  5893  AATCCCACTGACCTTTG  5909
```

Range 2: 6046 to 6122 GenBank Graphics ▼ Next Match ▲ Previous Match First Match

Score	Expect	Identities	Gaps	Strand
140 bits(154)	1e-36	77/77(100%)	0/77(0%)	Plus/Plus

```
Query  138   TACTCTTCCAACCCAAGAGGAGATTGAAAATCTTCCTGCCTTCCCTCGGGAAAAACTGAC  197
             ||||||||||||||||||||||||||||||||||||||||||||||||||||||||||||
Sbjct  6046  TACTCTTCCAACCCAAGAGGAGATTGAAAATCTTCCTGCCTTCCCTCGGGAAAAACTGAC  6105

Query  198   TCTGCGTCTCTTGCTGG  214
             |||||||||||||||||
Sbjct  6106  TCTGCGTCTCTTGCTGG  6122
```

Case 2

ATGATTTTTGGATACCAGAAACAAGTTTCATACTTACTATTATAGTTGGAATATTTCTGGTTGTTAC
AATCCCACTGACCTTTGTACTCTTCCAACCCAAGAGGAGATTGAAAATCTTCCTGCCTTCCCTCG
GGAAAAACTGACTCTGCGTCTCTTGCTGG

Homo sapiens ROS proto-oncogene 1, receptor tyrosine kinase (ROS1), transcript variant 3, mRNA

Sequence ID: NM_001378902.1 Length: 8451 Number of Matches: 2

Range 1: 5826 to 5910 GenBank Graphics ▼ Next Match ▲ Previous Match

Score	Expect	Identities	Gaps	Strand
154 bits(170)	4e-41	85/85(100%)	0/85(0%)	Plus/Plus

```
Query  1     ATGATTTTTGGATACCAGAAACAAGTTTCATACTTACTATTATAGTTGGAATATTTCTGG  60
             ||||||||||||||||||||||||||||||||||||||||||||||||||||||||||||
Sbjct  5826  ATGATTTTTGGATACCAGAAACAAGTTTCATACTTACTATTATAGTTGGAATATTTCTGG  5885

Query  61    TTGTTACAATCCCACTGACCTTTG  85
             ||||||||||||||||||||||||
Sbjct  5886  TTGTTACAATCCCACTGACCTTTG  5909
```

Range 2: 6046 to 6122 GenBank Graphics ▼ Next Match ▲ Previous Match ▲ First Match

Score	Expect	Identities	Gaps	Strand
140 bits(154)	8e-37	77/77(100%)	0/77(0%)	Plus/Plus

```
Query  85    TACTCTTCCAACCCAAGAGGAGATTGAAAATCTTCCTGCCTTCCCTCGGGAAAAACTGAC  144
             ||||||||||||||||||||||||||||||||||||||||||||||||||||||||||||
Sbjct  6046  TACTCTTCCAACCCAAGAGGAGATTGAAAATCTTCCTGCCTTCCCTCGGGAAAAACTGAC  6105

Query  145   TCTGCGTCTCTTGCTGG  161
             |||||||||||||||||
Sbjct  6106  TCTGCGTCTCTTGCTGG  6122
```

Case 3

TGAATATAGTGGAATCAGTGAGAATATTATATTAGTTGGAGATGATTTTTGGATACCAGAAACAA
GTTTCATACTTACTATTATAGTTGGAATATTTCTGGTTGTTACAATCCCGCTGACCTTTGTACTCTTC
CAACCCAAGAGGAGATTGAAGATCTTCCTGCCTTCCCTCGGGA

Homo sapiens ROS proto-oncogene 1, receptor tyrosine kinase (ROS1), transcript variant 3, mRNA

Sequence ID: NM_001378902.1 Length: 8451 Number of Matches: 2

Range 1: 5785 to 5910 GenBank Graphics ▼ Next Match ▲ Previous Match

Score	Expect	Identities	Gaps	Strand
224 bits(247)	9e-62	125/126(99%)	0/126(0%)	Plus/Plus

```
Query  1     TGAATATAGTGGAATCAGTGAGAATATTATATTAGTTGGAGATGATTTTTGGATACCAGA  60
             ||||||||||||||||||||||||||||||||||||||||||||||||||||||||||||
Sbjct  5785  TGAATATAGTGGAATCAGTGAGAATATTATATTAGTTGGAGATGATTTTTGGATACCAGA  5844

Query  61    AACAAGTTTCATACTTACTATTATAGTTGGAATATTTCTGGTTGTTACAATCCCGCTGAC  120
             |||||||||||||||||||||||||||||||||||||||||||||||||||||| |||||
Sbjct  5845  AACAAGTTTCATACTTACTATTATAGTTGGAATATTTCTGGTTGTTACAATCCCACTGAC  5904

Query  121   CTTTG  126
             |||||
Sbjct  5905  CTTTG  5909
```

Range 2: 6046 to 6096 GenBank Graphics ▼ Next Match ▲ Previous Match ▲ First Match

Score	Expect	Identities	Gaps	Strand
88.7 bits(97)	5e-21	50/51(98%)	0/51(0%)	Plus/Plus

```
Query  126   TACTCTTCCAACCCAAGAGGAGATTGAAGATCTTCCTGCCTTCCCTCGGGA  176
             |||||||||||||||||||||||||||| ||||||||||||||||||||||
Sbjct  6046  TACTCTTCCAACCCAAGAGGAGATTGAAAATCTTCCTGCCTTCCCTCGGGA  6096
```
